# Usefulness of TI-scout images in the assessment of late gadolinium enhancement in children

**DOI:** 10.1186/s12968-021-00719-2

**Published:** 2021-03-18

**Authors:** Badr Bannan, Julien Aguet, Aswathy Vaikom House, Navjot Gill, Vivian P. Tassos, Afsaneh Amirabadi, Mike Seed, Christopher Z. Lam, Shi-Joon Yoo

**Affiliations:** 1 Department of Diagnostic Imaging, The Hospital for Sick Children, University of Toronto, 555 University Avenue, Toronto, ON M5G1X8 Canada; 2 Department of Pediatrics, Division of Cardiology, Labatt Family Heart Centre, The Hospital for Sick Children, University of Toronto, Toronto, ON Canada

**Keywords:** Late gadolinium enhancement, Magnetic resonance, Myocardium, Fibrosis, TI-scout, Look-Locker

## Abstract

**Background:**

Cardiovascular magnetic resonance (CMR) late gadolinium enhancement (LGE) requires identification of the normal myocardial nulling time using inversion time (TI)-scout imaging sequence. Although TI-scout images are not primarily used for myocardial assessment, they provide information regarding different signal recovery patterns of normal and abnormal myocardium facilitating identification of LGE in instances where standard LGE images alone are not diagnostic. We aimed to assess the diagnostic performance of TI-scout as compared to that of standard LGE images.

**Methods:**

CMR studies with LGE imaging in 519 patients (345 males, 1–17 years) were reviewed to assess the diagnostic performance of LGE imaging in terms of the location of LGE and the pathologic entities. The diagnostic performance of the TI-scout and standard LGE imaging was classified into four categories: (1) equally diagnostic, (2) TI-scout superior to standard LGE, (3) standard LGE superior to TI-scout, and (4) complementary, by the consensus of the two observers.

**Results:**

The study cohort consisted of 440 patients with negative LGE and 79 with evidence for LGE. For a negative diagnosis of LGE, TI-scout and standard LGE images were equally diagnostic in 75% of the cases and were complementary in 12%. For patients with LGE, TI-scout images were superior to standard LGE images in 52% of the cases and were complementary in 19%. The diagnostic performance of TI-scout images was superior to that of standard LGE images in all locations. TI-scout images were superior to standard LGE images in 11 of 12 (92%) cases with LGE involving the papillary muscles, in 7 /12 (58%) cases with subendocardial LGE, and in 4/7 (57%) cases with transmural LGE. TI-scout images were particularly useful assessing the presence and extent of LGE in hypertrophic cardiomyopathy (HCM). TI-scout was superior to standard LGE in 6/10 (60%) and was complementary in 3/10 (30%) of the positive cases with HCM.

**Conclusions:**

TI-scout images enhance the diagnostic performance of LGE imaging in children.

## Background

Cardiovascular magnetic resonance (CMR) late gadolinium enhancement (LGE) in has become a gold standard technique for non-invasive assessment of myocardial infarction and fibrosis [1]. LGE imaging requires precise prescription of the inversion time (TI) when the signal of the normal myocardium is nulled > 10 min after the injection of a gadolinium-based contrast agent. The TI nulling time of normal myocardium is typically assessed using a modified Look-Locker TI-scout sequence (MOLLI) [1, 2]. Standard LGE imaging is then performed to produce optimal contrast between the healthy and pathologic myocardium using the identified TI nulling time. Although TI-scout imaging is not primarily used for clinical myocardial assessment, it provides valuable information regarding the kinetics of gadolinium in normal and abnormal myocardium [2, 3]. In our day-to-day practice, we found that TI-scout images are frequently helpful or even played a primary role in the identification of abnormal myocardial regions, especially when LGE is mild or heterogeneous, or involved the subendocardial or subepicardial region.

Based on our clinical experience, we hypothesized that TI-scout imaging enhanced the diagnostic performance of LGE imaging in children. In this retrospective study, we aimed to compare the diagnostic performance of the TI-scout images with that of the standard LGE images in the identification of abnormal myocardium.

## Methods

**Fig. 1:**
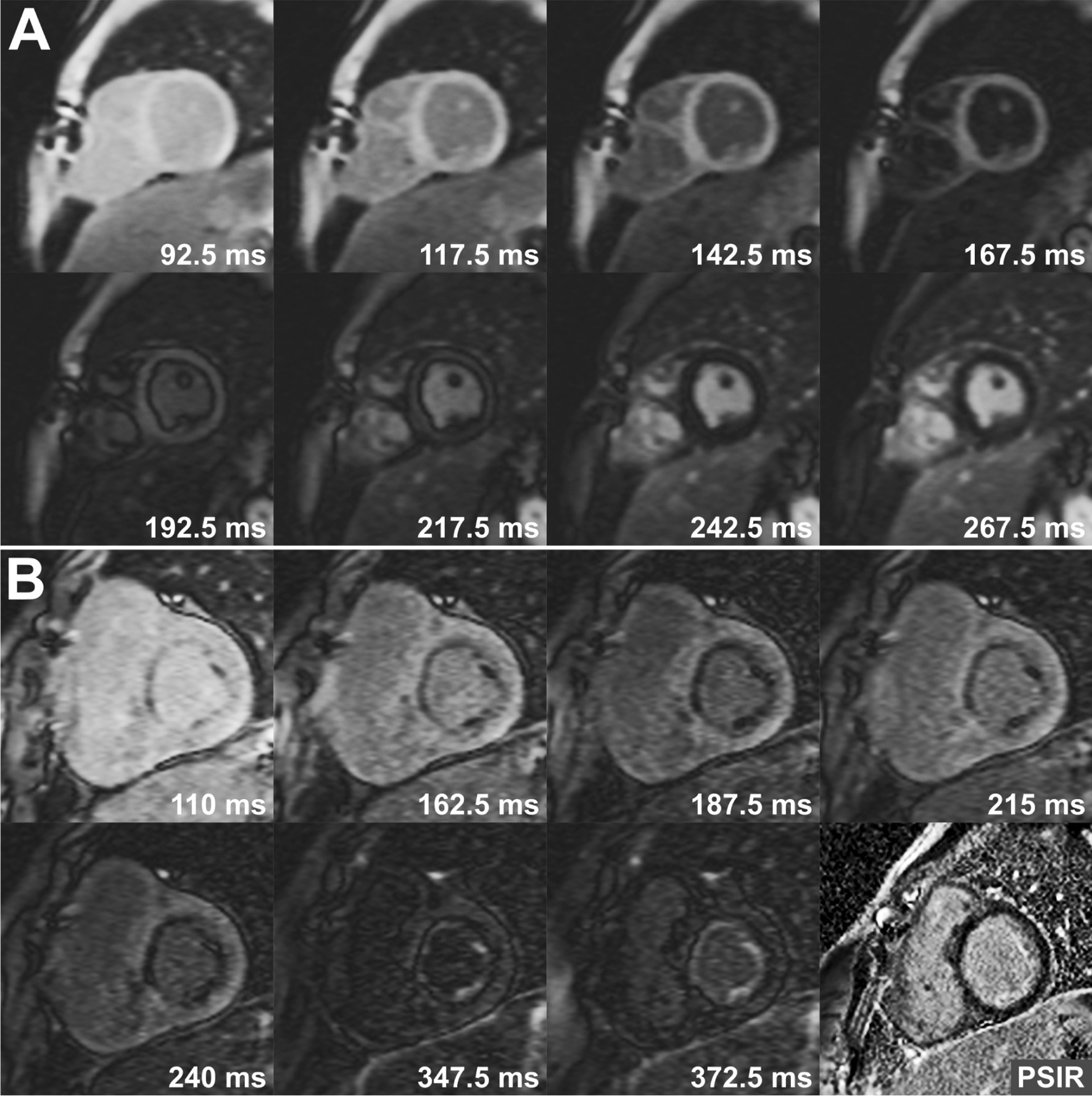
TI-scout images showing different signal intensity recovery after inversion pulse in normal (**a**) and abnormal (**b**) myocardium. In images obtained from a patient with aortic stenosis (**b**), there is obvious endocardial/subendocardial fibrosis in all TI-scout images with changing signal intensity. Note that late gadolinium enhancement (LGE) is barely identifiable in phase sensitive inversion recovery (PSIR) image in right lower corner

With the approval by our institutional Research Ethics Board, we identified 598 consecutive patients who underwent CMR with LGE imaging between January 2016 and December 2017. Among them, we excluded 79 patients whose TI-scout and standard LGE images were not optimum for proper assessment of LGE. The reasons for non-diagnostic quality included artefact from body motion or irregular breathing pattern (79%), poor patient’s cooperation related to medication such as adenosine and atropine (21%), metallic artefact (6%) and others. We retrospectively assessed the images and medical records of the remaining 519 cases (345 males, 1–17 years). All CMR was performed on a 1.5 CMR system (Avanto, Siemens Healthineers, Erlangen, Germany) using the parameters shown in Table 1. LGE was performed approximately 10 min after an intravenous injection of 0.3 mMol/kg of gadobenate dimeglumine (n = 83) or 0.2 mMol/kg of gadobutrol (n = 336) at (Fig. 1). TI-scout images were acquired in mid-ventricular short-axis plane using a MOLLI sequence. Standard LGE images were acquired in multiple short-axis and axial planes using segmented FLASH (fast low angle shot magnetic resonance imaging) and single-shot balanced steady state free precession (bSSFP) sequences. Additional TI-scout images were also obtained in multiple short-axis, 4-chamber and 2-chamber planes in patients with suspected LGE at standard LGE images.

**Table 1:** Technical parameters

Parameter	Modified Look-Locker TI-scout	Standard LGE (segmented PSIR-FLASH)	Standard LGE (single-shot PSIR-bSSFP)
ECG gating	Throughout R–R interval	Mid-late diastole	Mid-late diastole
ECG triggering	Every 2 R–R intervals	Every 2 R–R intervals	Every 2 R–R intervals
Breath-hold	No	Yes, if cooperative	No
Acceleration factor	2	No	2
Field of view	250–350 mm	250–350 mm	250–350 mm
Slice thickness	7 mm	6 mm	6 mm
Pixel size	1.3 × 1.3 mm	1.3 × 1.3 mm	1.8 × 1.8 mm
Flip angle	30°	45°	40°
TR/TE	2.93/1.25 ms	3.04/1.26 ms	2.57/1.09 ms
Signal averages	1	1 for breath-hold, 3 for uncooperative patients	1

*ECG* electrocardiographic, *FLASH* fast low angle shot, *PSIR* phase-sensitive inversion recovery, *bSSFP* balanced steady-state free precession

The presence, location and extent of LGE were analyzed by comparing the findings seen in the pairs of TI-scout and standard LGE images obtained in the corresponding imaging planes. Postoperative fibrosis such as LGE of the right ventricular (RV) outflow tract in repaired tetralogy of Fallot and equivocal LGE of the RV insertion point to the ventricular septum were not regarded as positive findings. When LGE was identified, its location was classified as subepicardial, mid-myocardial, subendocardial, transmural, papillary muscles and mixed locations. The positive or negative diagnosis of LGE was made by reviewing all available images. The diagnostic performance of TI-scout and LGE imaging was graded as: (1) equally diagnostic, (2) TI-scout superior to standard LGE, (3) standard LGE superior to TI-scout, or (4) complementary for final diagnosis, with the knowledge of the positive or negative diagnosis of LGE for each case. “Complementary” was defined as the situation where the final diagnosis was able to be reached more convincingly by reviewing the images from both sequences than by reviewing the images of one sequence alone. The diagnosis of LGE, the location of LGE and the diagnostic performance of the LGE sequences were assessed qualitatively. In those cases where the perception and grading were difficult, the decision was reached after reassessment and consensus between the two observers (BB and SJY).

All statistical analyses were performed using SPSS (version 26.0, Statistical Package for the Social Sciences, International Business Machines, Inc., Armonk, New York, USA). Descriptive analysis was performed for demographic and clinical variables. For quantitative variables, data were expressed as median (range). Dichotomous variables were expressed as n (%). Comparison between groups were performed using non-parametric tests (Chi-square). A P-value of less than 0.05 (2-sided) was considered statistically significant.

## Results

The study cohort consisted of 519 patients, 440 with negative LGE and 79 with LGE. The patient characteristics are summarized in Tables 2 and 3. The diagnostic performance of the TI-scout and standard LGE is summarized in Tables 4, 5 and 6.

**Table 2:** Patient demographics

Parameters	Distribution
Age, median (range), years	11 (0–17)
Sex	Male: 345 (66.5%)
Weight median (range), kg	35.5 (2.5–139)
Body surface area, median (range), m^2^	1.19 (0.18–2.86)
General anaesthesia for CMR, n (%)	145 (27.9%)

**Table 3:** Disease categories

Category	LGE negative	LGE positive	Total
Unrepaired congenital heart diseases	44	6	50 (10%)
Repaired congenital heart diseases	223	25	248 (48%)
Aortic valvular diseases	19	3	22 (4%)
Anomalous coronary arteries	8	0	8 (2%)
Hypertrophic cardiomyopathy	22	10	32 (6%)
Cardiomyopathy or myocarditis (excluding hypertrophic cardiomyopathy)	86	31	117 (23%)
Heart transplantation	3	0	3 (1%)
Cardiac tumors	6	1	7 (1%)
Systemic vascular diseases	29	3	32 (6%)
Total	440 (85%)	79 (15%)	519

*LGE* late gadolinium enhancement

**Table 4:** Diagnostic performance of TI-scout versus standard late gadolinium enhancement in all cases

	Equally diagnostic	TI-scout superior to standard LGE	Standard LGE superior to TI-scout	Complementary	Total
LGE negative	330 (75%)	18 (4%)	40 (9%)	52 (12%)	440 (84.8%)
LGE positive	9 (11%)	41 (52%)	14 (18%)	15 (19%)	79 (15.2%)
Total	339 (65%)	59 (11%)	54 (10%)	67 (13%)	519 (100%)

*LGE* late gadolinium enhancement, *TI* inversion time

**Table 5:** Diagnostic performance of TI-scout versus standard LGE images in LGE positive cases by disease categories

Disease category	Equally diagnostic	TI-scout superior to standard LGE	Standard LGE superior to TI-scout	Complementary	Total
Unrepaired congenital heart diseases	1 (17%)	3 (50%)	0	2 (33%)	6
Repaired congenital heart diseases	1 (4%)	18 (72%)	3 (12%)	3 (12%)	25
Aortic valvular diseases	0	3 (100%)	0	0	3
Hypertrophic cardiomyopathy	1 (10%)	6 (60%)	0	3 (30%)	10
Cardiomyopathy or myocarditis (excluding hypertrophic cardiomyopathy)	5 (16%)	10 (32%)	10 (32%)	6 (19%)	31
Cardiac tumors	0	0	1 (100%)	0	1
Systemic vascular diseases	1 (33%)	1 (33%)	0	1 (33%)	3
Total	9 (11%)	41 (52%)	14 (18%)	15 (19%)	79 (100%)

*LGE* late gadolinium enhancement, *TI* inversion time

**Fig. 2:**
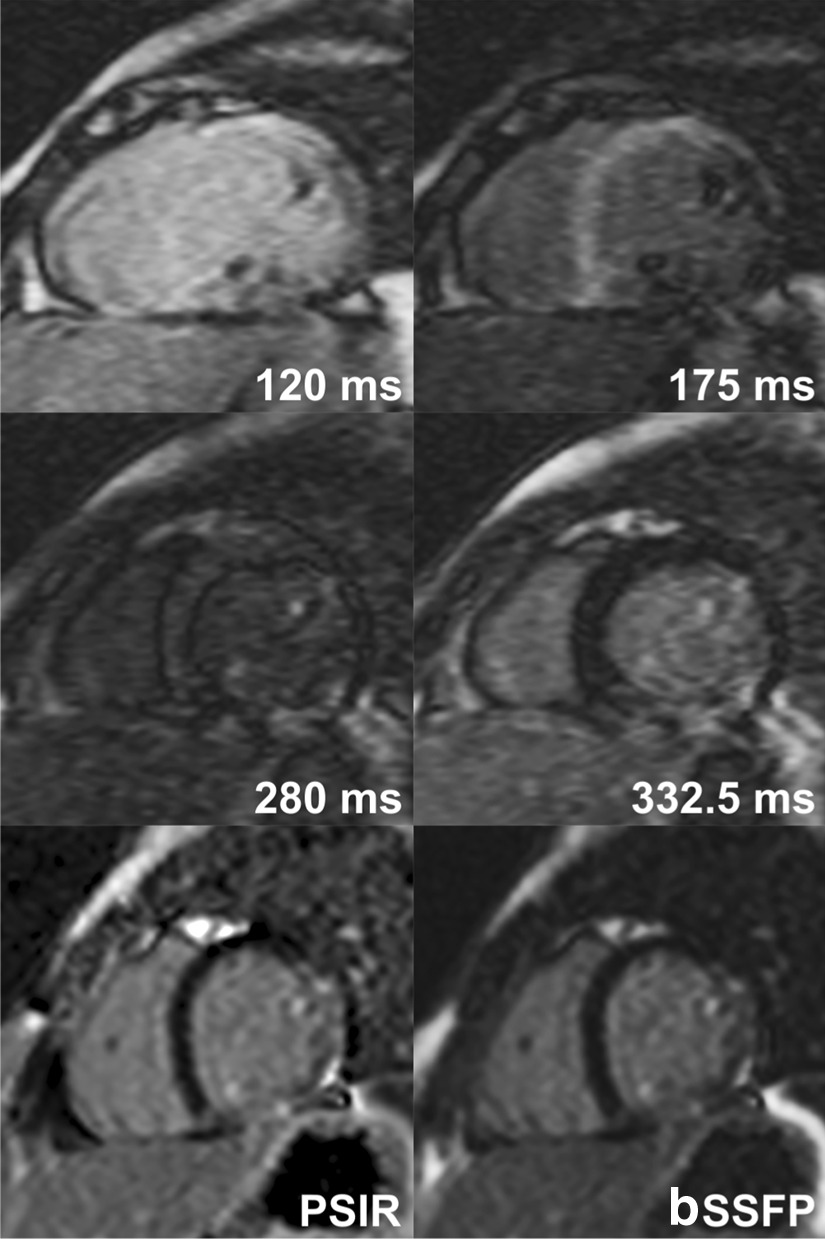
Late gadolinium enhancement (LGE) in a 13-year old with Duchenne muscular dystrophy. A similar extent of LGE involving subepicaridal/mid-myocardial layer of the inferolateral and lateral wall and papillary muscles of the left ventricle is seen in both TI (inversion time)-scout and standard LGE images, playing complementary roles in the diagnosis of LGE. Phase sensitive inversion recovery (PSIR) and balanced steady-state free precession (bSSFP) standard LGE images were obtained at TI of 330 ms

Among those without evidence for hyperenhancement on standard LGE imaging, TI-scout and standard LGE images were equally diagnostic in 75% of the cases and were complementary in 12% (Table 4). For those with evidence of LGE on standard LGE imaging, TI-scout images were superior to standard LGE images in 52% of the cases (P < 0.001) and they were complementary in 19% (Fig. 2). The standard LGE images were superior to TI-scout images in 9% of the cases with a negative diagnosis and 18% of those with a positive diagnosis. TI-scout images were more likely to be of incremental value in positive LGE group as opposed to negative LGE group [OR = 6.49, 95% CI 2.86–14.93, P < 0.001].

**Fig. 3:**
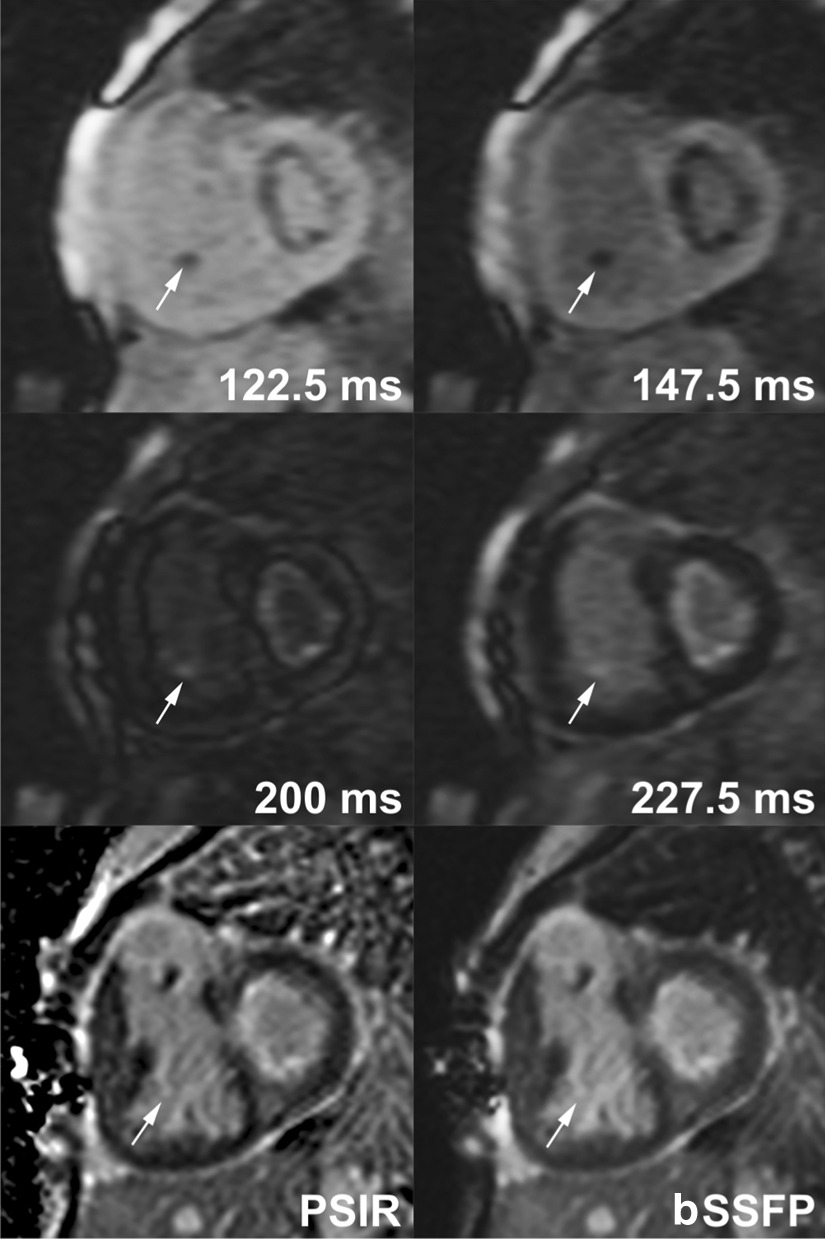
Late gadolinium enhancement (LGE) in a 6-month old with critical aortic and mitral stenosis. The presence and extent of LGE involving endocardium and subendocardium is appreciated better in TI (inversion time)-scout images than in phase sensitive inversion recovery (PSIR) and balanced steady state free precession (bSSFP) images. LGE of a papillary muscle (arrows) in the right ventricle is identifiable only in TI-scout images. Phase sensitive inversion recovery (PSIR) and balanced steady-state free precession (bSSFP) standard LGE images were obtained at TI of 280 ms

**Fig. 4:**
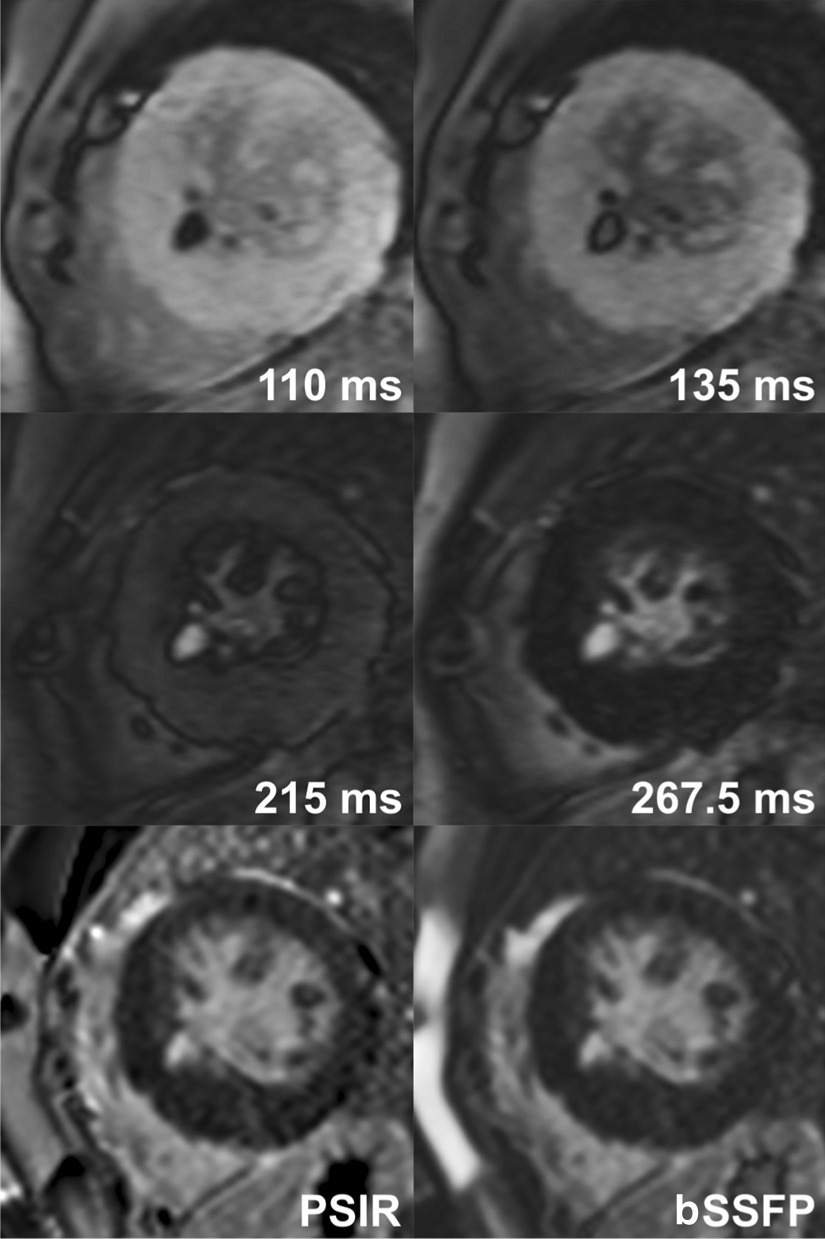
Focal subendocardial fibrosis in a 14-year old with concentric hypertrophic cardiomyopathy. A focal subendocardial fibrosis is well appreciated in TI (inversion time)-scout images. In phase sensitive inversion recovery (PSIR) and balanced steady-state free precession (bSSFP) images, high signal intensity involving the same region may represent either true late gadolinium enhancement (LGE) or blood pool in a myocardial crypt. Phase sensitive inversion recovery (PSIR) and steady-state free precession (SSFP) standard LGE images were obtained at TI of 310 ms

**Fig. 5:**
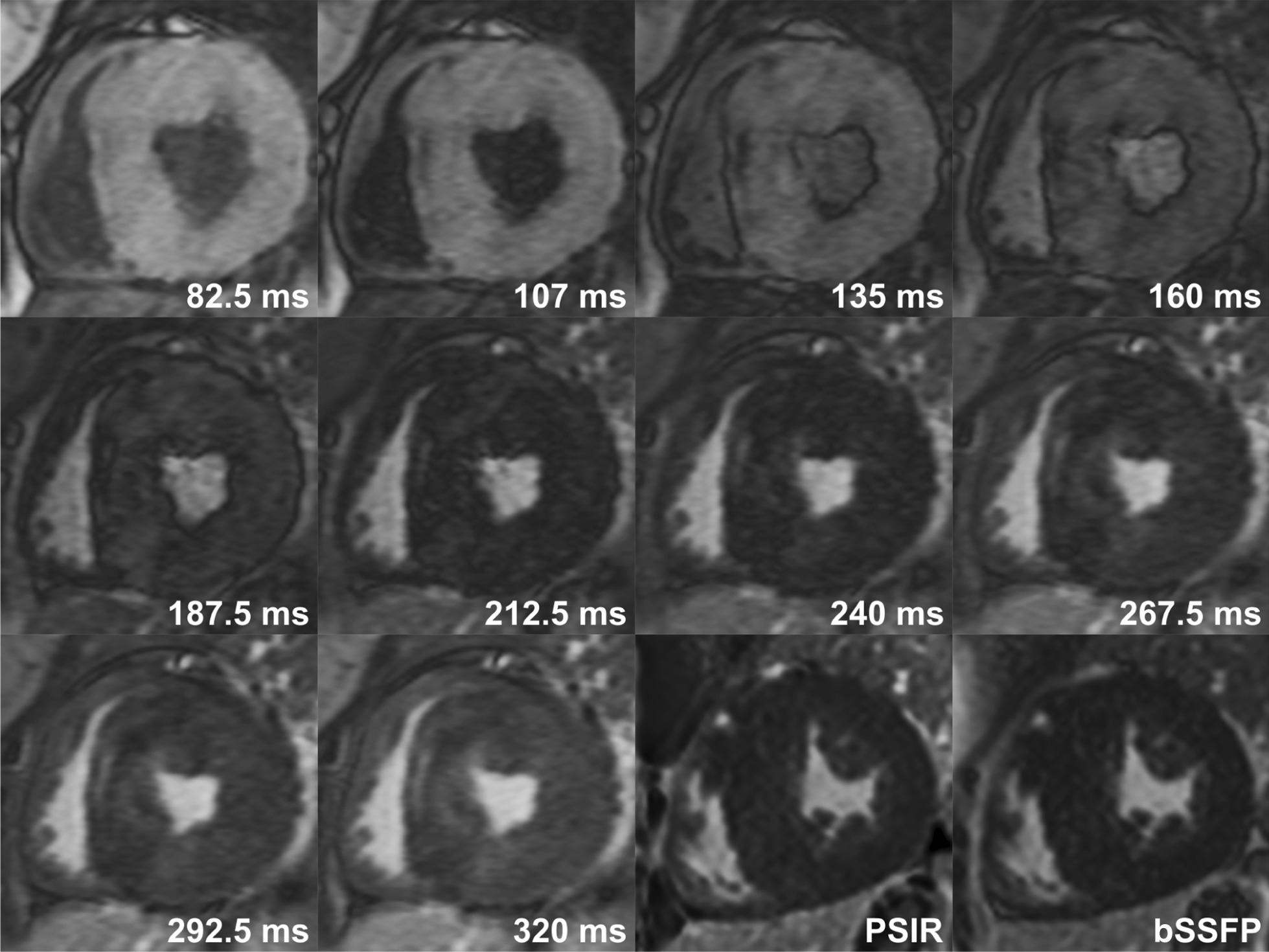
Heterogeneous mild late gadolinium enhancement (LGE) in a 16-year old with with hypertrophic cardiomyopathy. Heterogeneity of the signal intensity of the hypertrophic myocardium can be confidently appreciated only in TI (inversion time)-scout images. PSIR and bSSFP standard LGE images were obtained at TI of 280 ms

The diagnostic performance varied significantly according to the location of the LGE although TI-scout images were superior to standard LGE images in all locations (Table 6). TI-scout images were superior to standard LGE images in 11 of 12 (92%) cases with LGE involving the papillary muscles (Fig. 3), in 7 of 12 (58%) of the cases with endocardial-subendocardial LGE (Figs. 1 and 3), and in 4 of 7 (57%) cases with transmural LGE. TI-scout images were particularly useful in making the diagnosis of the presence and extent of LGE in hypertrophic cardiomyopathy (Figs. 4 and 5), aortic valvular disease and repaired congenital heart disease. TI-scout was superior to standard LGE in 6/10 (60%) and was complementary in 3/10 (30%) of the positive cases with hypertrophic cardiomyopathy (Figs. 4 and 5, Table 5). TI-scout images were superior to standard LGE images in cases involving the endocardium-subendocardial myocardium (58%) and papillary muscles (92%) that included most cases with repaired congenital heart disease or aortic valvular diseases.

**Table 6:** Diagnostic performance of TI-scout versus standard LGE images in LGE positive cases by location

LGE location	Equally diagnostic	TI-scout superior to standard LGE	Standard LGE superior to TI-scout	Complementary	Total
Subepicardium	4 (14%)	9 (32%)	8 (29%)	7 (25%)	28
Mid-myocardial	1 (6%)	7 (44%)	3 (19%)	5 (31%)	16
Endocardium and subendocardium	1 (8%)	7 (58%)	2 (17%)	2 (17%)	12
Transmural	1 (14%)	4 (57%)	1 (14%)	1 (14%)	7
Papillary muscle	1 (8%)	11 (92%)	0	0	12
Mixed	1 (25%)	3 (75%)	0	0	4
Total	9 (11%)	41 (52%)	14 (18%)	15 (19%)	79 (100%)

*LGE* late gadolinium enhancement, *TI* inversion time

## Discussion

LGE imaging is an established technique for detection of myocardial infarction and focal fibrosis [1]. However, there are limitations in detection of the lesions, particularly in areas of the myocardium adjacent to the blood pool and epicardial fat with standard LGE imaging. To overcome such limitations, the signal from the blood pool was completely or partially suppressed with or without T2 preparation between the inversion pulse and signal readout [4–7]. Although both complete and partial suppression techniques are helpful, neither technique is completely satisfactory. With complete suppression of the signal from the blood pool, localization of an enhancing lesion across the myocardium becomes difficult as the interface between the blood pool and myocardium is lost. Partial suppression technique can also be limited in detection of myocardial lesions with signal intensity similar to that of the partially suppressed blood pool. In addition, the differentiation between the slow flow artifact along the endocardial surface and the true subendocardial or endocardial enhancement can be challenging.

In LGE imaging, T1-scout sequence is routinely acquired to identify the optimal myocardial nulling time. The acquired images show the changes in the signal intensity over time, that can be plotted visually or graphically. Usefulness of TI-scout imaging in the assessment of cardiomyopathies such as amyloid infiltration and hypertrophic cardiomyopathy has been reported [2, 3]. In our experience in pediatric patients, we also found that these TI-scout images are helpful in determining the presence or absence of LGE when it is questionable or when LGE involves the endocardium, or subendocardial or subepicardial myocardium (Figs. 2 and 3). Based on our initial experience, we started to acquire additional TI-scout images along the planes where the standard LGE images demonstrated inconclusive LGE. This retrospective analysis of our experience demonstrated: (1) TI-scout and standard LGE images are equally diagnostic in excluding LGE, (2) TI-scout images are more often better than standard LGE in detecting LGE, (3) TI-scout and standard LGE images are often complementary in both negative and positive diagnosis of LGE, and (4) TI-scout images are particularly advantageous over standard LGE images in detecting the LGE involving the endocardium, subendocardial myocardium or papillary muscles, and LGE with low level enhancement such as so-called gray zone LGE or heterogeneous enhancement. The advantages of TI-scout in detection of LGE demonstrated in this study might not be well appreciated in pathologic conditions showing consistently strong LGE such as transmural myocardial infarction and, therefore, in adult population. Furthermore, we emphasize that TI-scout imaging does not replace the standard LGE imaging but that TI-scout images augment LGE image interpretation.

Endocardial fibroelastosis is an important diagnosis in the newborns with left ventricular outflow tract obstruction and hypoplasia. Although it is associated with poor prognosis, it can be removed surgically to provide the left ventricle with an opportunity to grow for later biventricular repair [8]. In this regard, LGE imaging has been advocated as a valuable tool for accurate identification of endocardial fibroelastosis [9, 10]. However, the sensitivity of LGE imaging for detection of endocardial fibroelastosis has not been established and even questioned [11]. In our study, TI-scout images were more diagnostic than standard LGE images in all 4 patients with endocardial fibroelastosis (Figs. 1 and 3). In standard LGE images, endocardial fibroelastosis did not stand out strongly against the bright blood pool unless LGE was distinctly strong. TI-scout images enabled the detection of LGE as they show the difference in gadolinium kinetics between normal myocardium, fibrotic myocardium and blood pool (Figs. 1 and 3). Similarly, TI-scout images were useful in clearly defining the transmural or subendocardial involvement of myocardial infarction.

LGE of the papillary muscle that was seen exclusively in patients with previous surgery in this study may represent fibrosis related to ischemia or chronic mechanical burden associated with atrioventricular valve regurgitation [12–15]. As the papillary muscles in the imaging plane may lack continuity with the remaining myocardium and are surrounded by the blood pool, LGE of the papillary muscles may escape detection with the standard LGE images alone because of the low contrast between the enhanced papillary muscles and blood pool. Therefore, complete or partial suppression of the blood pool signal was reported helpful in detection of LGE involving the papillary muscles [4, 6]. In our study, T1-scout images played the major role in the detection of LGE involving the papillary muscles (Fig. 3). In making the diagnosis of LGE of the papillary muscles, it is important to be sure that the enhancing structure is not the chordae tendinae that normally enhance in LGE imaging by cross-referencing the cine images.

In patients with HCM, identification of LGE is important for risk stratification and is associated with increased risk for sudden cardiac death or appropriate discharge of implantable cardioverter defibrillator as well as heart failure [16–18]. The fibrotic myocardium in HCM typically is easily identifiable as areas with high signal intensities. However, it may also show low level LGE in the periphery of the strongly enhancing region [19, 20]. Furthermore, the hypertrophic myocardium may show diffusely heterogeneous LGE without clearly identifiable margins. As the areas of low level LGE called gray zones may also serve as arrhythmogenic foci, proper assessment of the extent of the gray zones has been advocated [19, 20]. In our study as well as in Amano et al. study, the areas of low level or heterogeneous signal intensities were much easier to perceive in TI-scout images than in standard LGE images. At least in one of the multiple TI-scout images, heterogeneity of the signal intensity is obvious and any uncertainty in standard LGE images can be eliminated with confidence (Fig. 5).

## Study limitations

The study population was heterogeneous with the patients with various types of lesions, and the number of patients for each lesion was relatively small, which limited statistical analysis. The diagnostic performance of the TI-scout and standard LGE images were assessed qualitatively by visual comparison of the two sets of images but without any blinded assessment or pathological correlation. Lastly, utility of TI-scout in the assessment of the RV LGE was not able to be studied because of the small number of cases involving the RV. To assess the diagnostic impact of TI-scout images in assessing LGE in individual lesions, prospective quantitative studies are required using the pre-defined protocols including imaging parameters and planes in larger patient cohorts.

## Conclusions

TI-scout images enhance the diagnostic performance of LGE imaging by playing the primary role or complementing the weakness of standard LGE images in the assessment of myocardial infarction or fibrosis. Therefore, active utilization of TI-scout imaging is advised. We propose routine acquisition of TI-scout images in three short-axis planes, vertical long axis planes of the RV and left ventricle, and 4-chamber plane. This approach would extend the exam by 3–4 min, and should be considered in addition to the standard LGE imaging so as to increase the confidence in standard LGE interpretation.

## Data Availability

The datasets used and/or analysed during the current study are available from the corresponding author on reasonable request.

## Abbreviations

bSSFP: Balanced steady-state free precession
CMR: Cardiovascular magnetic resonance
ECG: Electrocardiographic
FLASH: Fast low angle shot
LGE: Late gadolinium enhancement
MOLLI: Modified Look-Locker inversion recovery
PSIR: Phase-sensitive inversion recovery
RV: Right ventricle/right ventricular
TI: Inversion time
